# Can Phlorotannins Purified Extracts Constitute a Novel Pharmacological Alternative for Microbial Infections with Associated Inflammatory Conditions?

**DOI:** 10.1371/journal.pone.0031145

**Published:** 2012-02-02

**Authors:** Graciliana Lopes, Carla Sousa, Luís R. Silva, Eugénia Pinto, Paula B. Andrade, João Bernardo, Teresa Mouga, Patrícia Valentão

**Affiliations:** 1 REQUIMTE/Laboratório de Farmacognosia, Departamento de Química, Faculdade de Farmácia, Universidade do Porto, Porto, Portugal; 2 CEQUIMED/Laboratório de Microbiologia, Departamento de Ciências Biológicas, Faculdade de Farmácia, Universidade do Porto, Porto, Portugal; 3 GIRM - Marine Resources Research Group, School of Tourism and Maritime Technology, Polytechnic Institute of Leiria, Peniche, Portugal; The City University of New York-Graduate Center, United States of America

## Abstract

Bacterial and fungal infections and the emerging multidrug resistance are driving interest in fighting these microorganisms with natural products, which have generally been considered complementary to pharmacological therapies. Phlorotannins are polyphenols restricted to brown seaweeds, recognized for their biological capacity. This study represents the first research on the antibacterial, antifungal, anti-inflammatory and antioxidant activity of phlorotannins purified extracts, which were obtained from ten dominant brown seaweeds of the occidental Portuguese coast.

Phlorotannins content was determined by the specific dimethoxybenzaldehyde (DMBA) method and a yield between 75 and 969 mg/Kg phloroglucinol units (dry matter) was obtained. *Fucus spiralis* ranked first, followed by three *Cystoseira* species. The anti-inflammatory potential of the purified extracts was assessed *via* inhibitory effect on nitric oxide (NO) production by lipopolysaccharide-stimulated RAW 264.7 macrophage cells, *Cystoseira tamariscifolia* being the one showing promising activity for the treatment of inflammation. NO scavenging ability was also addressed in cell free systems, *F. spiralis* being the species with highest capacity. The antimicrobial potential of the extracts was checked against five Gram-positive and four Gram-negative bacteria and three fungi strains, that commonly colonize skin and mucosa and are responsible for food contamination. The different extracts were more effective against Gram-positive bacteria, *Staphylococcus epidermidis* being the most susceptible species. Concerning antifungal activity, *Trichophyton rubrum* was the most sensitive species.

Although the molecular mechanisms underlying these properties remain poorly understood, the results obtained turn phlorotannins purified extracts a novel and potent pharmacological alternative for the treatment of a wide range of microbial infections, which usually also present an inflammatory component. In addition to the biological properties demonstrated herein, phlorotannins extracts may also be preferred, in order to avoid side effects and allergic reactions commonly associated with synthetic drugs.

## Introduction

**Figure 1:**
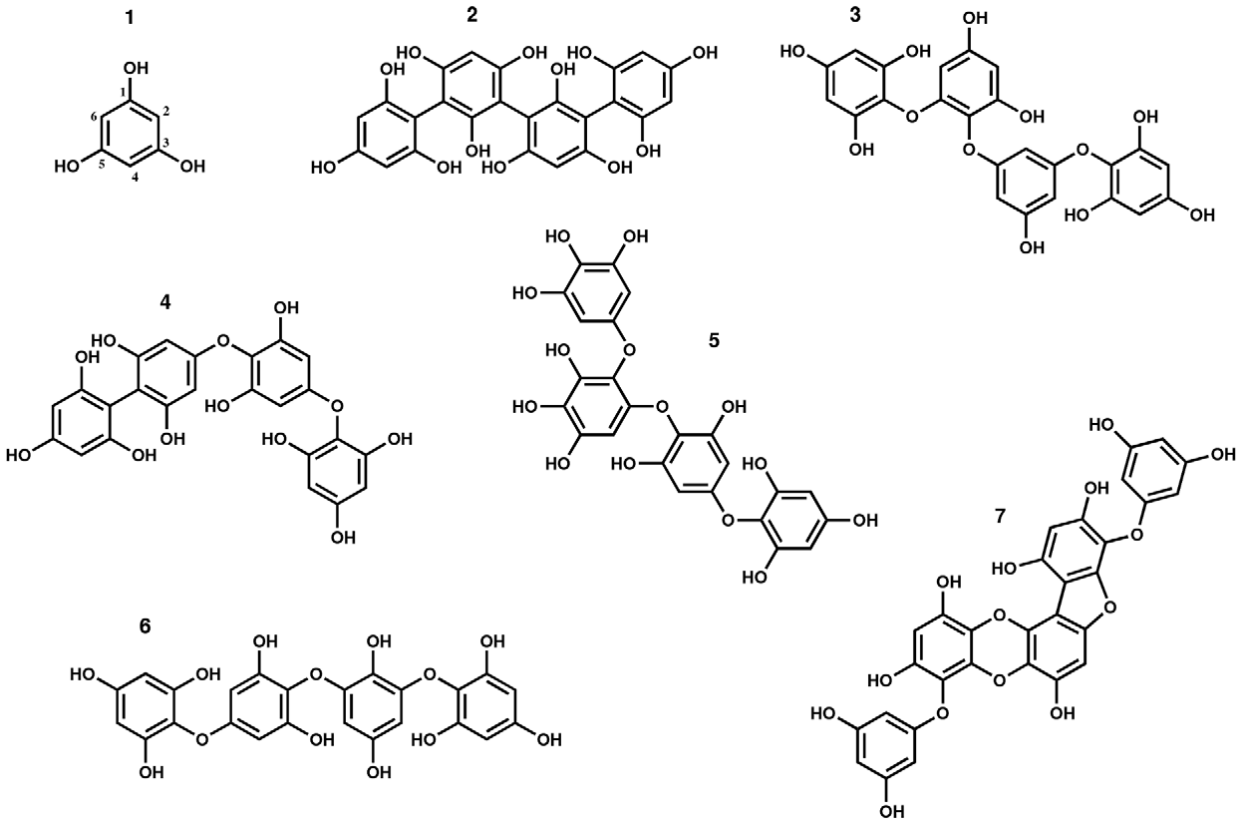
Chemical structures of different types of phlorotannins. Phloroglucinol (**1**), Tetrafucol A (**2**), Tetraphlorethol B (**3**), Fucodiphlorethol A (**4**), Tetrafuhalol A (**5**), Tetraisofuhalol (**6**), and Phlorofucofuroeckol (**7**).

Tannins are considered to be one of the most broadly distributed types of plants natural products [1]. These polyphenols are commonly divided into distinct groups according to their structures. They consist of flavonoids or gallic acid polymers in terrestrial plants, while in seaweeds they are composed of phloroglucinol (1,3,5-trihydroxybenzene) units. The last ones, known as phlorotannins, span a wide range of molecular sizes (from 126 Da to 650 kDa) and can be subdivided into six specific groups (fucols, phlorethols, fucophlorethols, fuhalols, isofuhalols and eckols) (Figure 1), characterized by differences in the nature of the structural linkages between phloroglucinol units and the number of hydroxyl groups present [1].

Phlorotannins are restricted to brown seaweeds (Phaeophyceae) and are biosynthesised through the acetate-malonate pathway in Golgi apparatus, in the perinuclear area of the cell. They are stored in vesicles called physodes, appearing as a colourless and refractive acidic fluid [2]. As they are not normally secreted outside the cell, it is necessary for the cells to be damaged before phlorotannins release. Phlorotannins concentration in brown seaweeds can vary among species, being affected by seaweed size, age, tissue type, salinity, season, nutrient levels, intensity of herbivory, light intensity and water temperature. Their concentration can reach the maximum in temperate and tropical Atlantic (up to 20% of brown seaweed dry mass), and the minimum in tropical Pacific and Indo-Pacific regions. Species from the order Fucales are richer in this kind of compounds [1], [3].

These compounds have primarily been regarded as defence chemical agents. Due to their protein precipitating capacity, they are capable of deterring grazing by herbivores, especially by fish. They are also important components of the cell wall and are responsible for the absorption of ultraviolet radiation [4].

Like other polyphenolic compounds, phlorotannins have been regarded as potential beneficial for human health [5]. Nowadays, diverse properties of phlorotannins are reported on biological systems, namely anti-inflammatory [6], anti-allergic [7], anti-viral [8], anti-cancer [9], bactericide [10], antioxidant [6], anti-diabetic activities and also radioprotective effects [11].

In particular, the anti-inflammatory potential of phlorotannins has attracted attention. The effect of phlorotannins extracts or isolated compounds from Laminariaceae on the arachidonic dependent pathway inflammatory response was reported [6], [12], [13]. A few studies regarding the effects of seaweeds extracts on nitric oxide (NO) production by cells involved in the inflammatory response have been performed [6], [14]. NO is an important inflammatory mediator synthesized from arginine by nitric oxide synthase (NOS). It is a diffusible free radical with many functions in diverse biological systems. It plays an important role as a vasodilator, neurotransmitter and in the immunological system, as a defence against tumour cells, parasites and bacteria. However, under pathological conditions, and when an oxidative environment takes place, an isoform of NOS is initiated. This isoform is known as inducible nitric oxide synthase (iNOS), and is responsible for the over production of NO [15]. Overproduction of nitric oxide is involved in the pathogenesis of septic shock and subsequent multiple organ dysfunction. The induction of the iNOS gene has been proposed to be a major factor in pathological vassal dilatation and tissue damage [5].

Polyphenols interaction with bacterial enzymes and proteins may be the reason for their antimicrobial activity [16]. Under certain conditions, in order to avoid side effects and allergic reactions, natural antibiotics or antibacterial substances are preferred over synthetic ones. Besides this, infection usually determines a subsequent inflammatory process. Considering the emerging multidrug resistance and the natural products potential, the bactericidal activity of phlorotannins from the brown seaweed *Ecklonia kurome* has been previously evaluated against a range of pathogenic bacteria [10].

**Figure 2:**
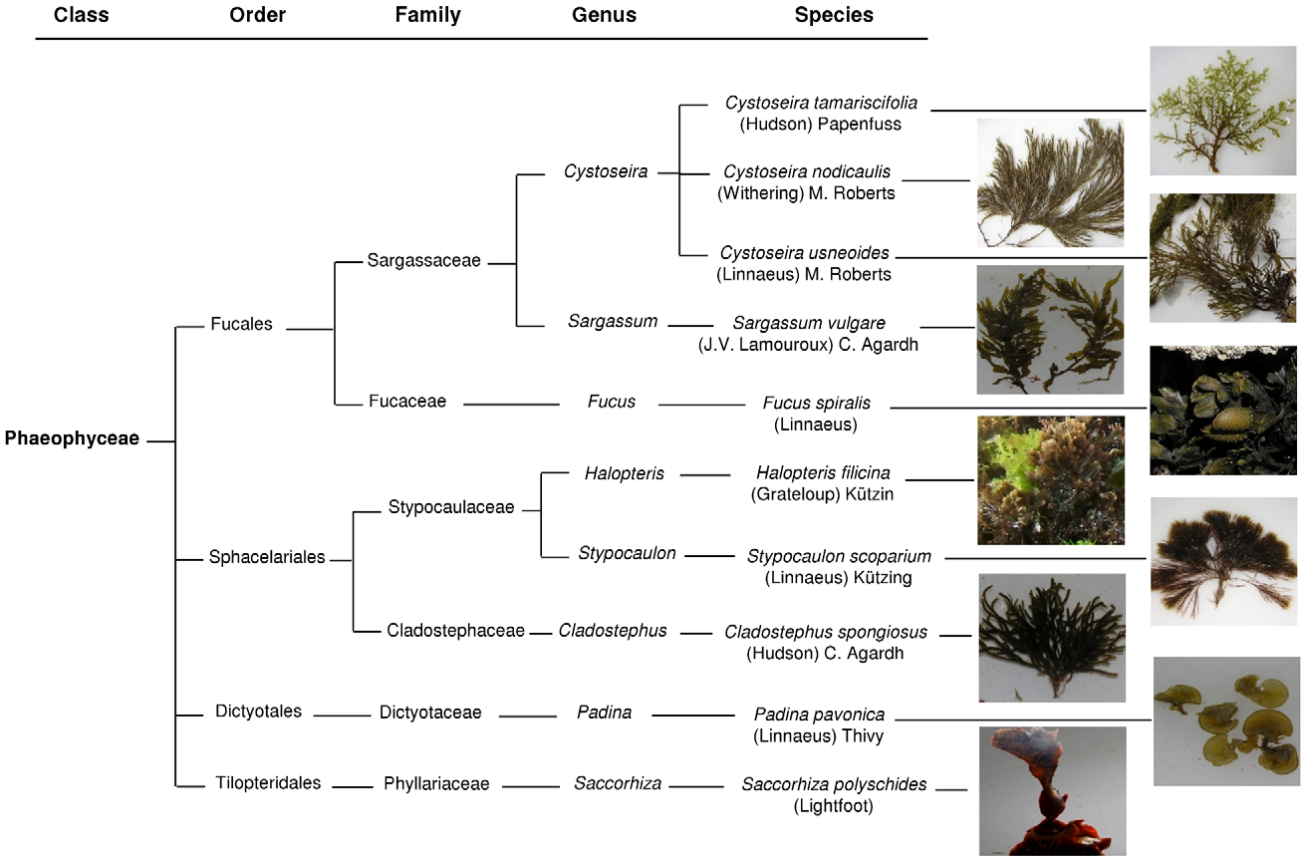
Phylogenetic representation of the seaweeds species studied.

This work aimed the evaluation of the *in vitro* anti-inflammatory, antioxidant, antibacterial and antifungal capacities of phlorotannins purified extracts for the first time. For this purpose ten species of Phaeophyta, belonging to eight genera of the orders Fucales, Sphacelariales, Dictyotales and Tilopteridales, not studied before, were collected along the Portuguese west coast (Figure 2). Additionally, the correlation of these biological properties with the seaweed species and their phlorotannins content was proposed.

## Materials and Methods

### Standards and reagents

Phloroglucinol, sodium pyruvate, thiazolyl blue tetrazolium bromide (MTT), β-nicotinamide adenine dinucleotide reduced form (NADH), sodium nitroprussiate dehydrate (SNP), sulphanilamide, toluene, methanol, dimethyl sulfoxide (DMSO), 2,4-dimethoxybenzaldehyde (DMBA) and lipopolysaccharide (LPS) from *Salmonella enterica* were purchased from Sigma-Aldrich (St. Louis, MO, USA). Cellulose microcrystalline for thin-layer chromatography, N-(1-naphthyl) ethylenediamine, acetone, hexane and acetic acid (glacial) were obtained from Merck (Darmstadt, Germany). Gentamicin was purchased from Applichem (Darmstadt, Germany) and fluconazole was kindly provided by Pfizer. Hydrochloric and *ortho*-phosphoric acid were purchased from Panreac (Barcelona, Spain).

Mueller Hinton Broth (MHB) and Mueller Hinton Agar (MHA) media were purchased from Liofilchem (Teramo, Italy) and Sabouraud dextrose agar (SDA) from Bio-Mèrieux (Marcy L'Etoile, France). Dulbecco's Modified Eagle Medium (DMEM), Dulbecco's phosphate buffered saline (DPBS), heat inactivated foetal bovine serum (FBS) and Pen Strep solution (Penicillin 5000 units/mL and Streptomycin 5000 µg/mL) were purchased from Gibco (Invitrogen, Paisley, UK). RPMI-1640 broth medium (with L-glutamine, without bicarbonate, and with the pH indicator phenol red) was purchased from Biochrom AG (Berlin). The murine macrophage-like cell line RAW 264.7 was purchased from Abcam (Cambridge, UK).Water was deionised using a Milli-Q water purification system (Millipore, Bedford, MA, USA).

### Sampling

Individuals of ten Phaeophyta species (Figure 2) were randomly collected in the coast of Peniche (west Portugal) and identified by Teresa Mouga, PhD (GIRM). Each seaweed sample corresponds to a mixture of 3–4 individuals in the same stage of development. With the exceptions of *Cystoseira usneoides* and *Cystoseira nodicaulis*, collected in 2009, all samples were collected in 2008. *Sacchorhyza polyschides* was collected in June, *C. usneoides* and *C. nodicaulis* in November, and the remaining species in September. After collection, samples were placed on ice and immediately transported to the laboratory in insulated sealed ice-boxes, to protect them from heat, air and light exposition. The fresh biomass was then cleaned, washed with NaCl aqueous solution (3.5% m/m) to remove epiphytes and encrusting material and kept at −20°C, prior to their lyophilisation in a Labconco 4.5 Freezone apparatus (Kansas City, MO, USA). The dried material was powdered and kept in the dark, in a desiccator, until it was subjected to phlorotannins extraction.

### Extracts preparation

The conditions used for extraction were adapted from those proposed by Koivikko and co-workers [4]. In order to maximize the efficiency of phlorotannins extraction, lipids were removed from aliquots of 0.5 g of powdered lyophilized material with n-hexane prior to the aqueous acetone extraction. The n-hexane treatment was repeated three times with 1 mL of solvent followed by centrifugation (5 minutes at 4000 rpm, 300× g) in a Rotofix 32 A Hettich centrifuge. After hexane removal, the defatted material was extracted four times with 10 mL of acetone∶water (7∶3), for 1 hour at 400 rpm, followed by centrifugation for 5 minutes at 4000 rpm. The organic fractions were combined and evaporated to dryness under reduced pressure, at 30°C.

### Extracts purification

The conditions used for extracts purification were adapted from those proposed by Fairhead and colleagues [17]. The dried acetone:water extract was resuspended in 30 mL of methanol, adsorbed into cellulose (approximately 2× the residue weight) and then dried under reduced pressure, at 30°C. This mixture was washed with toluene to remove pigments, until the filtrate run clear. Then, cellulose was rinsed with acetone∶water (7∶3) to release the phlorotannins. After centrifugation (5 minutes at 4000 rpm), the purified extract was evaporated to dryness under reduced pressure, at 30°C.

### Phlorotannins quantification (DMBA assay)

The quantification methodology was adapted from that proposed before [18]. The working reagent was prepared by mixing equal volumes of DMBA (2%, m/v) and hydrochloric acid (6%, v/v) prepared in glacial acetic acid. Aliquots of each extract (50 µL) were mixed with 250 µL of working reagent in a 96 wells plate. The reaction was conducted at room temperature, in the dark, during 60 minutes. The absorbance was determined at 515 nm in a Multiskan Ascent plate reader (Thermo Electron Corporation).

The quantitative determination of phlorotannins was carried out using phloroglucinol external standard. A series of seven phloroglucinol concentrations, ranging between 0.98 and 62.50 µg/mL, was used to obtain a linear calibration curve (y = 0.0254x) presenting an r^2^ value of 0.9998.

In order to evaluate phlorotannins recovery, phloroglucinol (98% purity) was used, as it was the only standard commercially available. Known amounts of phloroglucinol (0.250 and 0.500 mg) were added and the sample was treated under the conditions described for extraction and purification. The procedure was repeated 3 times and the total recovery of phloroglucinol was between 88 and 95%.

### Anti-inflammatory capacity

#### Cell culture and treatments

Murine macrophage RAW 264.7 cells were cultured at 37°C, in DMEM supplemented with 10% FBS and 2% Pen Strep solution in a humidified atmosphere of 5% CO_2_. Cells were inoculated at a density of 150 000 cells/well into 48 well plates and cultured until confluence.

Cells were pre-treated with different concentrations of phlorotannins purified extracts or vehicle for 1 hour. Following the addition of 1 µg/mL LPS (or vehicle) cells were further incubated for 18 h at 37°C in a humidified atmosphere of 5% CO_2_. The final concentration of DMSO was 0.5%. Four independent assays were performed in duplicate.

### Cell viability

#### Lactate dehydrogenase (LDH) assay

The LDH released into the culture medium was used as an index of cell death. After the incubation period of 18 h at 37°C the culture medium was carefully removed from each well and taken to determine the activity of LDH released by death cells. LDH activity was determined following the oxidation of NADH during the conversion of pyruvate to lactate at 340 nm. Results are expressed as LDH activity in the medium of exposed cells, relative to control, without extract [19].

#### Thiazolyl blue tetrazolium (MTT) assay

After the incubation period (18 h at 37°C), RAW 264.7 cells were washed with DPBS and then incubated for 30 minutes with MTT (0.5 mg/mL in DMEM). The yellow tetrazolium salt MTT is converted by mitochondrial dehydrogenases of metabolically active cells to an insoluble purple formazan product, which was then solubilized with DMSO. The extent of the reduction to formazan within the cells was quantified by measuring the absorbance at 510 nm [19].

#### NO production by RAW 264.7 cells

After the incubation period the nitrite accumulated in the culture medium was measured as an indicator of NO production [15]. Based on the Griess reaction, equal volumes of culture supernatant and Griess reagent [1∶1 mixture (v/v) of 1% sulphanilamide and 0.1% N-(1-naphthyl) ethylenediamine in 2% H_3_PO_4_] were mixed and incubated for 10 min in the dark, at room temperature.

The absorbance of the chromophore formed during the diazotization of nitrite with sulphanilamide and subsequent coupling with naphthylethylenediamine dichloride was read at 562 nm. Control values were obtained in the absence of phlorotannins purified extracts and after addition of LPS.

### Nitric oxide scavenging capacity

Nitric oxide was generated from sodium nitroprussiate dehydrate and measured by the Griess reagent as before [20]. SNP (6 mg/mL) in phosphate buffer was mixed with different concentrations of phlorotannins purified extracts and incubated at room temperature for 1 hour under light. Griess reagent was added and absorbance was read at 562 nm as previously described. Control values were obtained in the absence of phlorotannins purified extracts. Four independent assays were performed in duplicate.

### Antimicrobial activity

#### Microorganisms

The study included nine bacteria species [*Staphylococcus aureus* (ATCC 20231), *Staphylococcus epidermidis* (ATCC 20044), *Salmonella typhimurium* (ATCC 43971), *Proteus mirabilis* (ATCC 4479), *Escherichia coli* (ATCC30083), *Pseudomonas aeruginosa* (ATCC 50071), *Bacillus cereus* (ATCC 31), *Enterococcus faecalis* (ATCC 20477) and *Micrococcus luteus* (ATCC 20030)] and three fungi species [*Candida albicans* (ATCC 10231), *Aspergillus fumigatus* (ATCC 46645) and *Trichophyton rubrum* (CECT 2794)]. Cultures were obtained from the Department of Microbiology, Faculty of Pharmacy, Porto University (Portugal). *Candida krusei* (ATCC 6258) and *S. aureus* (ATCC 25923) were used for quality control in antifungal and antibacterial assays, respectively. All microorganisms were stored in broth medium with 20% glycerol at −70°C and sub-cultured in SDA for fungi and MHA for bacteria before each test, to ensure optimal growth conditions and purity.

### Antibacterial effect

Minimum inhibitory concentration (MIC) was determined by employing broth microdilution methods based on the Clinical and Laboratory Standards Institute (CLSI) guidelines, reference documents M07-A8 and M100-S19, with minor modifications [21]. Briefly, the suspensions of bacteria cultures were prepared in ampoules containing 2 mL of NaCl 0.85% suspension medium (api®, Biomérieux, Marcy l'Étoile, France). After adjusting the turbidity to 0.5 McFarland, suspensions were diluted in MHB till the final bacterial density of 1.5×10^6^ CFU/mL.

The MIC of phlorotannins purified extracts was determined by two-fold serial dilution method, in 96-well plates. The initial concentration was 31.3 mg/mL dry matter, for each tested species. Briefly, 50 µL of the bacterial suspension was added in each well, which contained 50 µL of phlorotannins purified extract dilutions in MHB medium. The maximum DMSO concentration didn't exceed 2.5% (v/v). The plates were incubated at 37°C in a humidified atmosphere containing 5% CO_2_, without agitation, for 18–24 h for both Gram positive and Gram negative bacteria.

The MIC was determined as the lowest concentration of phlorotannins purified extracts inhibiting the visual growth of the test culture on the microplate. Gentamicin MIC for *S. aureus* (ATCC 25923) was determined as quality control, and the result was within the recommended limits [21]. Sterility and positive controls in MHB medium alone and with 2.5% of DMSO (v/v) were included. Positive control wells contained microorganisms without antibiotics. The experiments were performed in duplicate and repeated independently three times, yielding essentially the same results.

### Antifungal effect

Antifungal assay was performed according to CLSI guidelines, reference documents M27-A3 for yeasts and M38-A2 for filamentous fungi, with minor modifications [22]. The MIC of phlorotannins purified extracts was determined by two-fold serial dilution method. Dilutions were prepared in RPMI-1640 broth, starting from 31.3 mg/mL dry matter for each extract. The solutions and cell suspensions in the test medium were then distributed into sterile 96-well plates. Maximum DMSO concentration didn't exceed 2.5% (v/v). The plates were incubated at 35°C in a humid atmosphere, without agitation, for 48 h for *C. albicans* and *A. fumigatus*, and at 30°C, for 96 h, for *T. rubrum*.

MICs were the lowest concentrations resulting in 100% growth inhibition. Fluconazole MIC for *C. krusei* (ATCC 6258) was determined as quality control, and the result was within the recommended limits [22]. Sterility and growth controls in RPMI-1640 medium alone and with 2.5% of DMSO (v/v) were included. The experiments were performed in duplicate and repeated independently three times.

### Determination of the minimum lethal concentration (MLC)

MLC of phlorotannins purified extracts was also accessed. The MLC was determined after 18–24 h (for both Gram positive and Gram negative bacteria), 48 h (for *C. albicans*) and 96 h (for *T. rubrum*) of incubation, by removing 20 µL of the contents from all wells showing no visible growth to SDA plates for fungi and to MHA plates for bacteria. The plates were incubated at 37°C for Gram positive and Gram negative bacteria, at 35°C for *C. albicans*, and at 30°C for *T. rubrum*. The MLC was defined as the lowest concentration showing 100% growth inhibition.

### Statistical analysis

Data were analysed by using SPSS software (Statistical Package for the Social Sciences) (version 18 for Windows). One-way analysis of variance (one-way ANOVA), using the Dunnett Multiple Comparison test, was carried out on data obtained from four assays performed in duplicate for each sample. A level of statistical significance at p<0.05, p<0.01 and p<0.001 was used.

## Results and Discussion

### Determination of phlorotannins content

The methodology used was previously applied to the obtainment of rich purified phlorotannins extracts. Despite being exhaustive, the extractive process guarantees the extraction of almost all free phlorotannins [4]. The purification step with cellulose is selective for tannins and allows the separation of phlorotannins from other compounds present in the extract [17].

Phlorotannins possess a unique structure (Figure 1) that is not found in terrestrial plants [12]. The quantification of phlorotannins in the purified extracts was done by DMBA colorimetric method using phloroglucinol as standard [18]. DMBA reacts specifically with 1,3- and 1,3,5-substituted phenols, such as phloroglucinol and phlorotannins, forming triphenylmethane pigments after electrophilic substitution [23]. This allows the indirect measurement of phlorotannins content, with good repeatability and high precision. Even so, the chromophore formed with DBMA depends on the phlorotannin class, i. e., ether bonds or aryl-aryl bonds react differently. As the reaction is also time and temperature dependent [23], these factors were strictly controlled. In the analysed seaweeds, the amount of phlorotannins varied between each genus and among species of the same genus (Table 1). *Saccorhiza polyshides* contained the lowest amount of phlorotannins (36.68 mg/Kg) and *Fucus spiralis* the highest one (968.57 mg/Kg).

**Table 1:** Phlorotannins content of brown seaweeds.

Sample	Phlorotannins (mg/Kg)^1^
*Cladostephus spongiosus*	81.64 (1.57)
*Cystoseira nodicaulis*	516.24 (3.15)
*Cystoseira tamariscifolia*	815.82 (17.46) (50,59349441)
*Cystoseira usneoides*	288.20 (8.01)
*Fucus spiralis*	968.57 (10.89)
*Halopteris filicina*	101.97 (1.57)
*Padina pavonica*	53.31 (1.57)
*Saccorhiza polyschides*	36.68 (1.82)
*Sargassum vulgare*	74.96 (7.76)
*Stypocaulon scoparium*	126.73 (0.91)

1 Mean (Standard Deviation) of three determinations, expressed as phloroglucinol units.

In previous studies other authors have found that phlorotannins levels can reach as much as 20% in some Fucales species and 30% in Dictyotales (dry matter basis) [24]. The amounts found in this work were considerably lower (<0.1%, dry matter) (Table 1). The content of cell-wall bound phlorotannins is usually much lower than the amount of soluble/free phlorotannins [4]. The determination of phlorotannins content was performed in the soluble fraction of the algal matrix after an exhaustive purification technique, thus only free ones were determined in our work. However, the species and geographic origin of the samples can also explain these results: it is known that the total amount of phenolic substances in brown seaweeds in temperate seas is lower than in tropical ones [25]. These authors found that *Padina pavonica*, a Dictyotales collected in the Canary Islands contained 0.69% phenolic substances, while *F. spiralis* presented 2.17%. Another species, *F. vesiculosus*, contained 14.5% of phlorotannins in ethanolic crude extract using the DMBA method [23].

In this survey the Sargassaceae family was represented by four species: *C. nodicaulis*, *C. usneoides*, *C. tamariscifolia* and *Sargassum vulgare* (Figure 2). *S. vulgare* was the one containing lower amounts of phlorotannins when compared with the remaining species of this family. Phlorotannins belonging to fuhalols and fucophlorethols classes are reported to be the main components of this family [26]. In this work the total phlorotannins amount found in the purified extracts of each family was assessed for the first time and revealed to be significantly different: the Fucaceae rank on the top while the Phyllariaceae rank at the bottom.

### Cytotoxicity of phlorotannins

Prior to testing the samples for their anti-inflammatory activities the cytotoxicity of phlorotannins purified extracts was assessed by using MTT and LDH assays on RAW 264.7 cells. The extracts didn't reveal to be cytotoxic under the tested concentrations (0.5 to 8.35 mg/mL, dry matter). This finding can be important for the possible use of these extracts in topic pharmacological formulas with anti-inflammatory properties, as they are not cytotoxic in cell systems.

The reduction of MTT varied between 92 and 107% of control, indicating that cells were metabolically active. LDH assay, which measures the activity of LDH released from dead cells into the culture medium, indicated cells viability between 89% and 107% (data not shown). It was not possible to evaluate the effect of *F. spiralis* extract once, under the experimental conditions, precipitation of the extract was observed (solubility below 0.52 mg/mL). In fact, in order to test the same concentrations for all seaweeds, *F. spiralis* extract needed the addition of high percentages of DMSO to completely solubilize. As high concentrations of DMSO are not advisable in cell systems (maximum 0.5%), the sample had to be much diluted and under those conditions no effect was observed.

Previously, phlorotannins rich extracts of *F. vesiculosus* were shown to lack any relevant toxic effects in a rat model [14]. Phlorotannins isolated from *Ecklonia cava* (7-phloroeckol, 6,6′-bieckol, phloroglucinol, eckol, fucodiphloroethol, phlorofucofuroeckol A and dieckol) also proved to have no cytotoxicity up to 100 µM in different cell lines (MRC-5, RAW264.7 and HL-60) [27].

### Inhibitory effect on cell NO production of phlorotannins purified extracts

Macrophages play an important role in inflammatory pathologies, being related to an overproduction of inflammatory mediators, such as NO. Under pathological conditions and in response to LPS, macrophage iNOS is induced, leading to NO overproduction, which may be important in the pathogenesis of various inflammatory conditions. Therefore, NO inhibitors are relevant for the prevention of inflammatory diseases [5].

**Figure 3:**
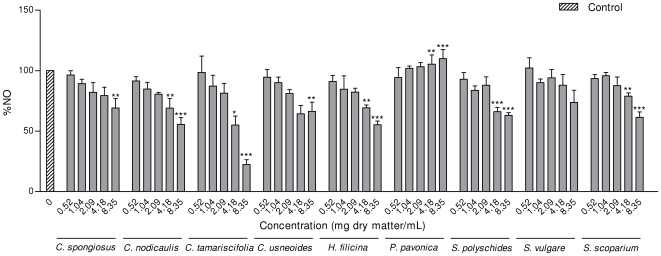
Effect of phlorotannins purified extracts on LPS-induced NO production in RAW 264.7 cells. Values of each group are expressed as % of LPS-only exposed cells (mean ± SEM of 4 independent assays). *p<0.05, **p<0.01, ***p<0.001.

As can be seen in Figure 3, phlorotannins purified extracts of most species were able to significantly reduce the levels of nitric oxide in LPS-exposed macrophage cells, at concentrations that are not cytotoxic. The only exceptions were *P. pavonica* and *S. vulgare*, which didn't significantly reduce NO levels in comparison with control cells.

Our results showed that *C. tamariscifolia* displayed a remarkable potential for reducing NO production (Figure 3). At the highest concentration, NO production was reduced to 25% (p<0.001) of control cells. *C. nodicaulis*, *Halopteris filicina* and *S. scoparium* were the species with highest activity: *C. nodicaulis* and *H. filicina* lead to a decrease of NO production to 55% and *S. scoparium* to 61% for the highest concentration (p<0.001) (Figure 3). These results may turn seaweeds phlorotannins extracts attractive as anti-inflammatory agents. The inhibitory effect of the extracts on NO production cannot be attributed to cytotoxic effects, since they didn't decrease cell viability, as discussed above.

It is known that brown seaweeds can exert anti-inflammatory activity by processes other than decreasing NO production. For example, anti-inflammatory effect *via* the arachidonic dependent pathway (down regulation of prostaglandin E2 generation) was observed for an *E. cava* phlorotannins extract [13]. In addition, isolated phlorotannins inhibited lipoxygenases and phospholipase A_2_s activity [12]. In another assay moderate Cox-1 inhibition by several phlorotannins was observed, which may contribute for their anti-inflammatory capacity [9]. In fact, phlorotannins can affect NO levels by direct scavenging and/or by decreasing NO production through the action in the inflammatory signaling cascade or by inhibiting the enzymes involved in NO production [6], [13].

The differences in the anti-inflammatory activity can be explained, at least partially, by the total amount of phlorotannins present in the seaweeds extracts. In what concerns to extracts of the same species but with different phlorotannins contents, the work by Zaragozá and colleagues with *F. vesiculosus* extracts [14] showed that the potential to reduce NO is proportional to the phlorotannins content. Considering the probable similarity of the qualitative composition of the three *Cystoseira* species studied, our results seem to agree with those of Zaragozá and co-workers [14], as the capacity of these species to reduce NO production was proportional to their phlorotannins content (Table 1, Figure 3). However, the qualitative composition of the extracts may not be excluded, as species with similar phlorotannins content presented different activities [28], [29]. For example, *S. polyschides* was much more active than *P. pavonica*, even having lower phlorotannins content.

This suggests phlorotannins rich extracts as potential raw material for the development of new pharmaceutical formulas with anti-inflammatory properties.

### Nitric oxide scavenging activity

The decrease of NO concentration can be due to the interference with NO production by LPS-stimulated macrophages or to the scavenging of the produced NO. In order to test the later hypothesis, a cell free assay with a NO donor was performed. Under the tested concentrations *Cladostephus spongiosus*, *H. filicina*, *S. polyschydes* and *P. pavonica* had no activity in the cell free system. As far as we know, the NO scavenging activity of phlorotannins purified extracts for the ten brown seaweeds collected was assessed for the first time.

**Figure 4:**
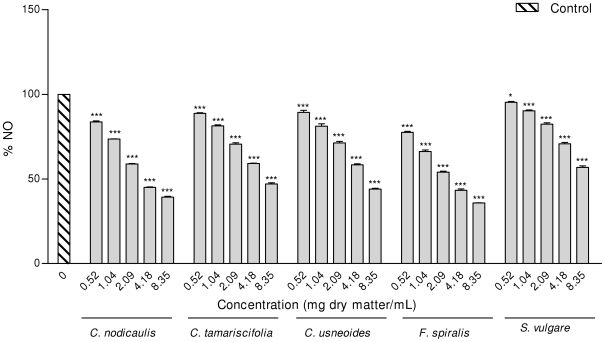
Effect of phlorotannins purified extracts on SNP-generated NO in a cell-free system. Values of each group are expressed as % of control (mean ± SEM of 4 independent assays). *p<0.05, **p<0.01, ***p<0.001.

Five of the brown seaweed species (*C. tamariscifolia*, *C. nodicaulis*, *C. usneoides*, *S. vulgare* and *F. spiralis*) showed significant nitric oxide scavenging activity, in a dose dependent manner (Figure 4). So, the decrease in NO seen in the cellular system may be partially attributed to direct NO scavenging, as the phlorotannins purified extracts of these species decreased the amount of nitrite generated from the decomposition of SNP. In contrast to the cellular assays, in which *C. tamariscifolia* showed the highest potential to reduce NO, in the cell free system *F. spiralis* and *C. nodicaulis* exhibited the highest capacity for NO radical scavenging, leading to a reduction of about 40% when compared with the control (Figure 4). On the other hand, the extracts of *H. filicina*, *S. polyschides* and *S. scoparium* reduced the levels of NO on cells stimulated by LPS, having no activity in the cell-free system. Again, the differences observed between the cellular and non-cellular assay suggest that besides NO scavenging, other mechanisms of decreasing NO may be involved in the cellular assay, for example those related to the several inflammatory cascade steps [6], [7], [13]. As so, the scavenging effect observed in the cell free system with *C. nodicaulis*, *C. tamariscifolia*, *C. usneoides* and *S. vulgare* can contribute to the decrease of NO noticed in the cellular system with these species.

### Antibacterial activity

The antibacterial activity of phlorotannins purified extracts from the studied species has been investigated *in vitro* for the first time herein. Extracts were tested against several Gram-positive and Gram-negative bacterial strains and MIC and MLC values were determined (Table 2). In a general way, Gram-positive bacteria were more sensitive to phlorotannins purified extracts than Gram-negative ones. The phlorotannins purified extracts showed bacteriostatic activity against all of the studied strains and bactericidal activity against five of them. There was no considerable variation in the MLC found among the different studied bacteria, with the exception of *M. luteus* treated with *F. spiralis* extract, which revealed to be the most lethal agent (Table 2). Overall, *S. epidermidis* was the most susceptible species among Gram-positive bacteria.

**Table 2:** MIC and MLC of phlorotannins purified extracts against selected bacteria.

	Gram-positive										Gram-negative							
Seaweed Extract^1^	*S. aureus*		*S. epidermidis*		*M. luteus*		*E. faecalis*		*B. cereus*		*P. mirabilis*		*E. coli*		*P. aeruginosa*		*S. typhimurium*	
	MIC	MLC	MIC	MLC	MIC	MLC	MIC	MLC	MIC	MLC	MIC	MLC	MIC	MLC	MIC	MLC	MIC	MLC
*C. spongiosus*	>31.3	-	31.3	>31.3	>31.3	-	>31.3	-	>31.3	-	>31.3	-	>31.3	-	>31.3	-	>31.3	-
*C. nodicaulis*	7.8	>31.3	3.9	31.3	15.6	31.3	>31.3	-	15.6	>31.3	15.6	>31.3	31.3	31.3	31.3	>31.3	31.3	>31.3
*C. tamariscifolia*	15.6	>31.3	7.8	>31.3	15.6	31.3	>31.3	-	15.6	>31.3	31.3	>31.3	>31.3	-	>31.3	-	>31.3	-
*C. usneoides*	15.6	>31.3	7.8	>31.3	31.3	>31.3	>31.3	-	31.3	>31.3	31.3	>31.3	>31.3	-	>31.3	-	>31.3	-
*F. spiralis*	7.8	>31.3	3.9	31.3	2.0	15.6	15.6	31.3	7.8	31.3	31.3	>31.3	>31.3	-	31.3	>31.3	>31.3	-
*H. filicina*	>31.3	-	31.3	>31.3	>31.3	-	>31.3	-	>31.3	-	>31.3	-	>31.3	-	>31.3	-	>31.3	-
*P. pavonica*	>31.3	-	15.6	>31.3	>31.3	-	>31.3	-	>31.3	-	>31.3	-	>31.3	-	>31.3	-	>31.3	-
*S. polyschides*	>31.3	-	15.6	>31.3	>31.3	-	>31.3	-	>31.3	-	>31.3	-	>31.3	-	>31.3	-	>31.3	-
*S. vulgare*	31.3	>31.3	7.8	>31.3	>31.3	-	31.3	>31.3	>31.3	-	31.3	>31.3	>31.3	-	>31.3	-	>31.3	-
*S. scoparium*	>31.3	-	31.3	>31.3	>31.3	-	>31.3	-	>31.3	-	>31.3	-	>31.3	-	>31.3	-	>31.3	-

1 mg/mL (dry matter).

According to the results obtained, *F. spiralis* and *C. nodicaulis* are the most promising seaweeds (Table 2).

### Gram-positive bacteria

In a general way, *Cystoseira* species and *F. spiralis* were the most active against *Staphylococcus*. *S. epidermidis* was the most sensitive species, with *F. spiralis* and *C. nodicaulis* presenting the lowest MIC and MLC values (Table 2). *S. aureus* was more sensitive to *C. nodicaulis* and *F. spiralis* (MIC = 7.8 mg/mL). *Staphylococcus* are Gram positive cocci. *S. aureus* and *S. epidermidis* are more often associated with human infection, *S. aureus* being the major cause of morbidity and mortality [30].

Of the studied seaweeds, *F. spiralis* presented the best activity against *M. luteus*, with a MIC value of 2.0 mg/mL (Table 2). This seaweed also presented the highest bactericidal activity, being capable of killing *M. luteus* at 15.6 mg/mL. *M. luteus* has been implicated in a variety of infections, including meningitis, endocarditis, septic arthritis and central nervous system infections in immunocompromised hosts [30].

In general, species belonging to the genus *Cystoseira* and *F. spiralis* were the ones with highest bacteriostatic and bactericidal activity, which can be related with their high content in phlorotannins.

### Gram-negative bacteria

Phlorotannins purified extracts evidenced lower activity against Gram-negative strains (Table 2). *C. nodicaulis* was the only seaweed with ability to inhibit *S. typhimurium* growth (Table 2). Most infections with *Salmonella* are caused by contaminated food and water. Of concern are *Salmonella* infections that are resistant to several antimicrobial agents, in which the serotype *Typhimurium* is included [31].

*P. mirabilis* and *E. coli* are commonly found in human gastrointestinal tract [31]. *P. mirabilis* was the most sensitive strain studied. It was inhibited by five of the studied extracts between 15.6–31.3 mg/mL (Table 2). *C. nodicaulis* was the most effective seaweed against this bacterium, and the only presenting bacteriostatic and bactericidal activity against *E. coli* (Table 2).

The physical differences between Gram-negative and Gram-positive bacteria can be the basis of the observed behaviour of phlorotannins extracts. Gram-negative bacteria are surrounded by an external membrane with high lipopolysaccharide content. This membrane, in addition to other mechanisms, confers bacteria the capacity to resist to several antibiotics and, consequently, to the action of many natural extracts [32].

Nagayama and co-workers [10] concluded that the antibacterial effects of phlorotannins tend to increase with the polymerization of phloroglucinol. *S. aureus* and *B. cereus* were the only Gram-positive strains studied by this group. *S. aureus* was more sensitive than *B. cereus*, which is in accordance with our results.

Attending to our data, species belonging to the genus *Cystoseira* and *Fucus* are probably the ones with phlorotannins of higher molecular weight, as they are the ones presenting lower MICs for all the studied bacteria. On the other hand, and according to the tannic compounds ability of reacting with amine groups of proteins by the their free hydroxyl groups [16], it is also probable that species from the genus *Cystoseira* and *Fucus* tend to have higher amount of phlorotannins with more hydroxyl groups in the free form. Among *Cystoseira* genus, although *C. tamariscifolia* presented the highest phlorotannins amount (Table 1), the extract was less effective against bacteria than that of *C. nodicaulis* and *C. usneoides*. Thus, it is possible that polyphenols present in *C. tamariscifolia* exhibit lower molecular weight or fewer hydroxyl groups free to react when compared with those in *C. nodicaulis* and *C. usneoides*.

Although the mechanism of action of phlorotannins is still obscure, the interaction of phlorotannins with bacterial proteins may play an important role [10]. Taking into account the MIC values obtained for *F. spiralis* and *C. nodicaulis*, these species appear to be promising for a future antimicrobial approach.

### Antifungal activity

Results of the antifungal screening are presented in Table 3. Phlorotannins extracts seemed to be less effective against fungi than bacteria. The purified extracts displayed antifungal properties against two fungi strains: *T. rubrum* and *C. albicans*.

**Table 3:** MIC and MLC of phlorotannins purified extracts against selected fungi.

	Fungi					
Seaweed Extract^1^	*C. albicans*		*A. fumigatus*		*T. rubrum*	
	MIC	MLC	MIC	MLC	MIC	MLC
*C. spongiosus*	>31.3	-	>31.3	-	>31.3	-
*C. nodicaulis*	7.8	>31.3	>31.3	-	3.9	7.8
*C. tamariscifolia*	>31.3	-	>31.3	-	31.3	>31.3
*C. usneoides*	31.3	>31.3	>31.3	-	7.8	31.3
*F. spiralis*	31.3	>31.3	>31.3	-	3.9	31.3
*H. filicina*	>31.3	-	>31.3	-	>31.3	-
*P. pavonica*	>31.3	-	>31.3	-	>31.3	-
*S. polyschides*	>31.3	-	>31.3	-	>31.3	-
*S. vulgare*	15.6–31.3	>31.3	>31.3	-	7.8	31.3
*S. scoparium*	>31.3	-	>31.3	-	>31.3	-

1 mg/mL (dry matter).

Under the tested conditions, *A. fumigatus*, commonly responsible for allergic symptoms associated with inhalation of spores [33], was resistant to the action of all extracts (Table 3).

Of the studied fungi, *T. rubrum* was the most sensitive to the phlorotannins purified extracts. *F. spiralis* and *C. nodicaulis* were the most effective species (MIC = 3.9 mg/mL), followed by *C. usneoides* and *S. vulgare* (MIC = 7.8 mg/mL), and *C. tamariscifolia* (MIC = 31.3 mg/mL). All other seaweed extracts were inactive under the tested concentrations (Table 3). The MLC was also assessed against *T. rubrum*. Best results were obtained with *C. nodicaulis*, being lethal for the fungi at 7.8 mg/mL. These results point *C. nodicaulis* as very promising for the future development of antimycotic drugs since the tested phlorotannins extract presented fungistatic and fungicidal activity. Dermatophytes are keratinophilic fungi normally found growing in the dead keratinized tissue of the stratum corneum of the skin, within and around the scalp hair and in the nails. *T. rubrum* is the principal agent of nail infections, also known as onychomycosis. These infections are difficult to treat because of the slow growth of nails [33].

Only four species displayed fungistatic activity against *C. albicans* under the tested concentrations, *C. nodicaulis* being the most effective one (Table 3). *C. albicans* is a commensal yeast and, as such, colonizes the mucosa of the majority of healthy humans without causing tissue damage. However, as opportunistic pathogens, *Candida* species can establish disease in a variety of permissive circumstances: C*andida* cells can disseminate from mucosa and gut, being the origin of invasive infections. Oral and vaginal thrushes are very common even in individuals with slightly weakened immunity. Biofilm-forming capacity greatly increases the potency of *Candida* to convert from the commensal stage into a virulent pathogen [33].

In a general way, *C. nodicaulis* revealed to be the most effective species against the studied fungi. Although it was not lethal for *C. albicans* under the tested concentrations, it presented the lowest MICs for both *C. albicans* and *T. rubrum*, respectively, being also lethal for the dermatophyte.

In conclusion, this article provides evidence that phlorotanins purified extracts have the capacity to act as antimicrobial, antioxidant and anti-inflammatory agents. Species belonging to the genus *Cystoseira* and *Fucus spiralis* have potential for the development of medicines with anti-inflammatory and antimicrobial activities, especially against Gram-positive strains and dermatophytes, which revealed to be more sensitive to them. Additionally, the NO scavenging capacity observed in cell-free systems confirms the antioxidant potential of phlorotannins, which can contribute to the overall beneficial effects. These results point phlorotannins extracts as potential pharmaceutical resources for combating infections with multi-etiological causes, as the same extract can cover a wide range of microorganisms and has the potential to act on inflammatory states and oxidative environment, commonly associated with microbial infections.
